# Use of an Extra-Tympanic Membrane Electrode to Record Cochlear Microphonics with Click, Tone Burst and Chirp Stimuli

**DOI:** 10.3390/audiolres11010010

**Published:** 2021-03-01

**Authors:** Laura M. Coraci, Andy J. Beynon

**Affiliations:** Vestibular & Auditory EP Lab—Department Otorhinolaryngology, Radboud University Medical Center, Ph. Van Leijdenlaan 15, 6525EX Nijmegen, The Netherlands; lauramc@live.nl

**Keywords:** electrocochleography, cochlear microphonic, tympanic membrane electrode, auditory evoked potentials

## Abstract

This study determined electrocochleography (ECochG) parameter settings to obtain cochlear microphonics (CM) with less invasive flexible extra-tympanic membrane electrodes. In 24 adult normal-hearing subjects, CMs were elicited by presenting click stimuli at 100 dBnHL, tone bursts (2 kHz) and broadband (BB) CE-chirps^®^ LS (Interacoustics, Middelfart, Denmark), both at 80 dBnHL. Different high-pass filters (HPFs) (3.3 Hz and 100 Hz, respectively) were used to investigate response quality of the CM. CMs were successfully obtained in 92–100% with click-, 75–83% with 2 kHz tone burst- and 58–63% with CE-chirp^®^-LS stimuli. Click stimuli elicited significantly larger CM amplitudes compared to 2 kHz tone bursts and BB CE-chirp^®^ LS (Interacoustics, Middelfart, Denmark). No significant differences were found between the two different high-pass filter (HPF) settings. The present study shows that it is possible to obtain clear CMs with the flexible extra-tympanic membrane electrodes using click stimuli. In contrast to 2 kHz tone bursts and CE-chirp^®^ (Interacoustics, Middelfart, Denmark) LS, clicks show a significantly higher success rate and are the preferred stimuli to confirm the presence or absence of CMs.

## 1. Introduction

Auditory evoked potentials (AEPs) from the cochlea and the auditory nerve can be assessed objectively by electrocochleography (ECochG), revealing four basic components within the first 5 ms after stimulus onset: (1) the compound action potential (AP), which can be described as a reflection of the combined firing of thousands of cochlear nerve fibers and can be clinically interpreted as a measure of the actual auditory response or “hearing potential”; (2) the summating potential (SP), which reflects the nonlinear distortion from the outer hair cells (OHCs) [1]; (3) the auditory nerve neurophonic (ANN), reflecting the auditory nerve firing most likely to occur as a response to low frequency tones [2]; and (4) the cochlear microphonic (CM), which is a preneural reproduction of the acoustic signal that “mirrors” the movement of the basilar membrane, reflecting the spatial summation of transducer currents produced by a large number of OHCs [3]. The first ECochG measurements in humans during surgery were performed by Perlman and Case in 1941 [4]. In the sixties, the development of computer averaging algorithms enabled the first nonsurgical ECochG recordings under local anesthesia [5]. This transtympanic (TT) ECochG is a rather invasive procedure, requiring an insertion and middle-ear placement of a needle recording electrode through the tympanic membrane, usually carried out by a surgeon. However, in the eighties, the less invasive Brainstem Evoked Response Audiometry (BERA) became more popular in clinical audiology to objectively assess hearing thresholds since it covered a wider range of the auditory pathway from the cochlear nerve (including the AP) up to the level of the auditory brainstem. Although, for the diagnosis, evaluation or prognosis of hearing losses in specific patient groups, such as Meniere’s Disease (calculating the ratio between the SP and compound AP as an indicator for endolymphatic hydrops), ECochG was still applied [6].

Besides the fact that CMs, in contrast to compound APs, appeared to be not very useful in assessing hearing thresholds and were also more difficult to distinguish from artefacts, its clinical value was rather limited [1]. Nevertheless, during the last decades, the CM has been gaining more clinical interest in the assessment of specific auditory pathologies; it is suggested that this preneural response could be of importance in patient groups where one is specifically interested in the functioning of different cochlear structures, such as in patients with mitochondrial hearing disorders [7]. Other ECochG studies reported its application in patients with auditory neuropathy/dyssynchrony spectrum disorders (ANSD). Since ANSD is characterized by normal OHC function but with absent or a disturbed neural synchrony at the higher brainstem level, subjects with ANSD reveal abnormally increased CM amplitudes during ECochG in comparison to normal-hearing subjects. Thus, advocating for the application of CM recordings as part of the diagnostic protocol to confirm ANSD should be considered [8].

More recently, CM recordings seem to be promising for the assessment of intracochlear trauma of the OHCs during the insertion of a cochlear implant [9]. These recent developments contributed to a revival of ECochG applications in clinical settings. For a systematic review, see Trecca et al. [10].

Although both otoacoustic emissions (OAEs) and CMs assess OHC activity, the lower frequencies (e.g., 500 Hz) are more difficult to obtain with OAEs compared to CMs, suggesting that CMs may have a higher diagnostic value [11].

To date, most studies are using a relatively invasive transtympanic (TT)-ECochG setup by inserting the electrode through the tympanic membrane and placing it on the cochlear round window or promontory to obtain CMs for OHC diagnosing purposes [12]. An alternative to this invasive TT-ECochG is the application of an extra-tympanic (ET) recording electrode placement in the ear canal or near the tympanic membrane. Even though the response amplitudes of ET-ECochG are four times smaller compared to TT-ECochG responses due to the greater distance to the neural source [13], ET-ECochG is still preferred in clinical audiology due to its less invasive and more patient-friendly nature. Nowadays, most ET-ECochG responses are captured by using a “tiptrode”, an “eartrode” or a tympanic membrane (TM) electrode. “Tiptrodes” are insert phone plugs that are wrapped in gold foil, the latter functioning as a recording site, while “eartrodes” are placed in middle part of the ear canal. A TM electrode is a flexible silicon-shielded electrode that can be placed closer to, or even against, the tympanic membrane, thus closer to the source of the neural response. A within-subject experiment comparison of those two ET methods reported a significant difference between the two electrode positions: ECochG with a TM electrode (from now on called TM-ECochG) revealed significantly higher response amplitudes and better reproducibility due to its closer proximity to the cochlea compared to “tiptrodes” located in the ear canal [13]. However, in contrast to “tiptrodes”, placement of a TM electrode requires some practical skills of the clinician to carefully place the electrode as close as possible to the tympanic membrane.

The quality of ECochG recordings, i.e., peak amplitudes and latencies, is highly dependent on specific recording parameters (electrode positions, electrode brand and filter settings) and stimulation parameters (stimulus type, stimulus repetition rate and polarity). With respect to the ECochG parameter setting, there seems to be no common consensus on the specific frequency for the high-pass filter (HPF). A wide variety of HPFs are clinically used, varying from 5 [14,15] to 100 Hz [16,17]. Since recording and acquisition parameters vary between different clinics, it is therefore recommended to obtain normative ECochG values based on their local clinic-specific recording conditions [18].

The current study will investigate the clinical feasibility to use a flexible ET silicon TM electrode to acquire CMs in normal-hearing subjects, as (1) the TM-ECochG recording is less invasive compared to the TT variant, and (2) it leads to higher response peak-to-peak amplitudes compared to the application of the ET alternative, “tiptrode” recordings [13]. In contrast to the few previous TM-ECochG studies, mainly focused on just AP and SP recordings [19], this study will specifically focus on the acquisition of CM responses. Since chirp stimuli have been clinically implemented in clinical AEP recording systems in the last decade [20], our second aim is to investigate which of the different stimulus types, including the level specific chirp (CE-chirp^®^ LS) (Interacoustics, Middelfart, Denmark), is preferred for optimal CM response recordings.

## 2. Materials and Methods

### 2.1. Subjects

Twenty-four normal-hearing adult subjects (10 males, 14 females) with a mean age of 24.6 years ± 2.6 (range: 20–32 years) participated in this study. All participants had pure tone thresholds ≤20 dBnHL from 250 to 8000 Hz for both ears and did not have any oto-neurological history. All subjects read the information brochure and signed the informed consent before participation. Participation was completely voluntarily.

### 2.2. TM-ECochG Parameters

An AEP recording system (Interacoustics Eclipse II ^®^, Denmark) was used to measure the right ear of each subject. The impedances of all disposable surface electrodes were ≤5 kΩ. A recording-time window of 10 ms in length was used to capture and average the ECochG responses. Considering the stimulus travel time through the 26.6 cm silicon tube, all stimuli arrived at the tympanic membrane at exactly 0 ms. The system has an integrated digital filter, and the responses were preamplified 100,000 times.

Three different test protocols were used to obtain CMs in response to a click, 2 kHz tone burst and broad band (BB) CE-chirp^®^ LS. To facilitate a clear interpretation of consistent CM responses, all stimuli were presented at a loud acceptable presentation level (LAPL) stimulus intensity of 100 dBnHL (click, 0.1 ms) and 80 dBnHL (2 kHz 1-1-1 cycle tone burst and CE-chirp^®^ LS), respectively, after subjective equal loudness perception was confirmed for all three stimuli in a (pilot) loudness scaling experiment at chosen stimulation levels. A stimulus repetition rate of 87.1/s was used with two different high-pass filters (HPFs), i.e., 3.3 or 100 Hz, and a fixed low-pass filter of 3000 Hz. To confirm reproducibility, all TM-ECochG responses were recorded at least twice for each stimulus polarity and averaged for all condensation and rarefaction responses. According to current CM protocols [17], CMs were confirmed by two additional recordings with a clamped tube, confirming absent CM responses. For each recording, 1500 stimuli were presented for each stimulus type consisting of at least six recordings: at least 2 condensation and rarefaction responses each and 2 clamped conditions with a rarefaction polarity (arbitrarily chosen) were recorded with an HPF of 3.3 Hz. The same procedure was repeated with an HPF set at 100 Hz.

### 2.3. Procedure

Prior to the recordings, the right ear canal of each subject was inspected for outer ear and/or tympanic abnormalities and cleaned (cerumen), followed by conventional tonal audiometry to confirm normal hearing. Fz and Fpz scalp locations were prepared with chlorhexidine 0.5% in ethanol 70% and scrubbed with a mild abrasive gel to reduce skin impedance. Subsequently, disposable surface electrodes were covered with conductive electrode paste and placed on Fz (inverting electrode) and on middle-forehead/Fpz (ground). Table 1 and Table 2 summarize stimulus and recordings parameters.

**Figure 1:**
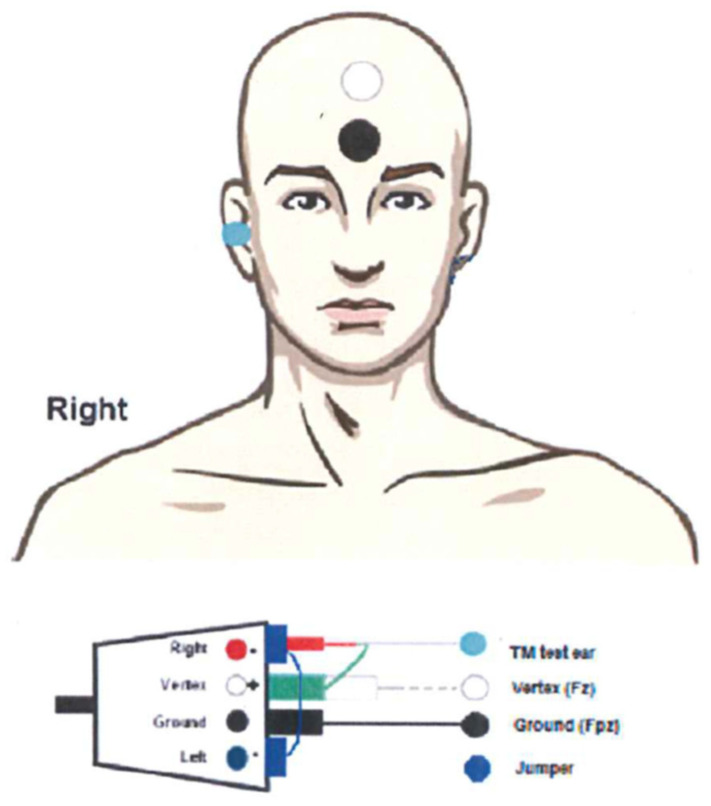
Electrode configuration using the TM electrode and EPA4 (with permission of Interacoustics, 2019).

The TM electrode (Sanibel, Denmark) was allocated in the AEP electrode interface (EPA4, Interacoustics) as inverting, according to standard “vertical montage” and connected to the jumper between left and right to allow single channel recordings (see Figure 1).

The patient was instructed to lie down comfortably on a bed on the left side (right ear up). To avoid any discomfort during electrode insertion, a puff of 10% xylocaine was sprayed in the right ear canal. In the meantime, the TM electrode was placed in a bath of saline and conductivity gel (Lectron II) with ratio 1:1 at room temperature for 10 min.

**Figure 2:**
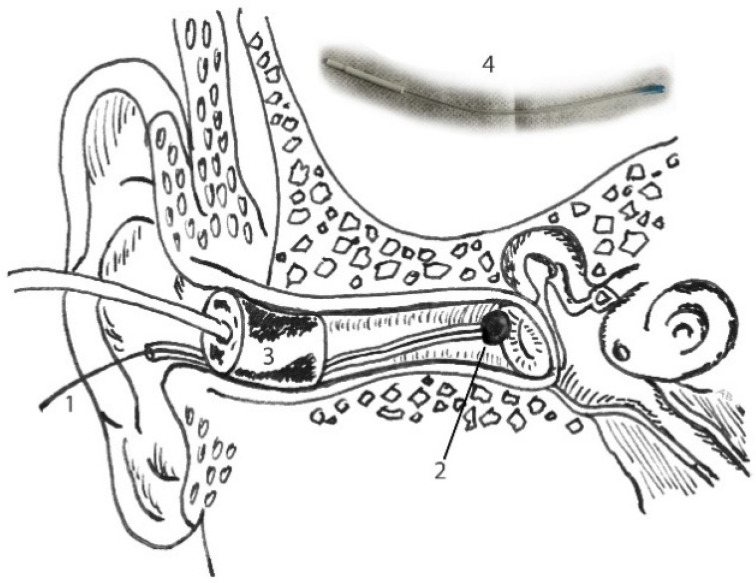
Placement of shielded tympanic membrane (TM) electrode with gel tip, fixed by placement of the insert probe: 1. Lead TM electrode (recording), 2. gel tip, directly placed against the superior half of the tympanic membrane, 3. insert probe (stimulus) and 4. inset: TM electrode (Sanibel ™).

Then, the ear canal was dried with a cotton bud, followed by TM-electrode placement at the superior half of the tympanic membrane, until low resistance was noticed. Lastly, an insert probe (E-A-RTONE 5A) was placed in the ear canal, simultaneously fixating the TM electrode (see Figure 2). Depending on the size of the individual ear canal, foam was cut approximately 2 mm around the tip, avoiding any obstruction or leakage of the soundwave. The subjects were instructed to lie down relaxed. When ECochG recordings were completed, a final ear canal inspection and cleansing of the ear canal were carried out. The whole experiment was performed in a soundproof and light-dimmed room and took about 75 min in total, including preparation time.

### 2.4. Statistical Analysis

Overall prevalence of the CM waveforms was calculated. CM was considered present when the responses from both polarities show an inversed phase, with an absence of the responses in the clamped-tube condition. The CM was defined as absent when both polarities were not reversible or reproducible or when a response was seen in the clamped tube condition.

Analysis using IBM SPSS Statistics version 21 (IBM SPSS, Inc., Chicago, IL, USA) was conducted to provide descriptive statistics for analysis of the success rate and means per stimulus type and HPF. Polarities were described individually. A repeated measures ANOVA design with planned contrasts was executed to analyze the difference in CM amplitude, CM latency and CM duration after changing the stimulus types (click, 2 kHz tone burst and CE-chirp^®^ LS) and HPF (3.3 Hz and 100 Hz). Levels of significance were defined at the 5% level (*p* < 0.05). Peak-to-peak amplitudes of CM sinusoidal response (nV) were used for analyses: for clicks and CE-chirps^®^ LS, amplitude was at the onset of first response; and for 2 kHz, amplitude was of the second sinusoid (1-1-1). CM latencies were defined as the time from the onset of the stimulus to the first CM response in milliseconds. CM durations were calculated from the onset of the first CM sinusoid until the end of the last CM sinusoid. Data was corrected when assumptions of normality and sphericity were violated. Differences between sexes and polarities were explored with one-way ANOVAs.

## 3. Results

### 3.1. CM Success Rates in Response to the Different Stimuli

TM-ECochG recordings of responses to acoustic stimuli were obtained in 24 normal-hearing adults. CMs were recognized in the recorded ECochG in response to a click stimulus (92–100%), a 2 kHz tone burst (75–83%) and a CE-chirp^®^ LS (58–63%): see Table 3.

**Figure 3:**
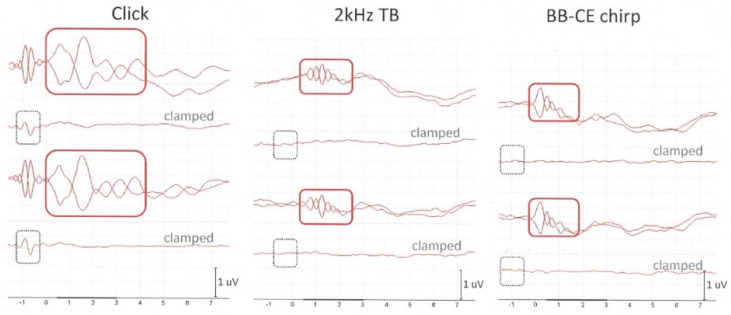
Example of a single subject showing from top to bottom: CMs (both polarities superimposed, 3.3 Hz HPF), the clamped tube condition (no CM), CMs (both polarities superimposed, 300 Hz HPF), clamped tube condition (no CM) evoked by a click, typical for all subjects (left graph), by a tone burst (atypical response: middle graph) and by a BB-CE chirp stimulus (atypical response: right graph). Note: in contrast to conventional lower stimulation rates used for SP/AP recordings, a higher stimulation rate was used to obtain CMs (87.1 Hz). Black rectangles: residual electrical stimulus artifact; red rectangle: CM response.

Typically, all subjects show clear CM responses evoked by a click: see Figure 3, left graph. An example of a subject with one of the best CM responses evoked by both sinusoidal stimuli (atypical) is shown in Figure 3 (middle and right graph). In most of the subjects, CM responses to tone bursts and CE-chirp^®^ LS were worse or hardly recognizable; i.e., they showed very low amplitudes.

### 3.2. Different Parameter Settings

Since normality of the sample mean distributions were violated (Shapiro-Wilk *p* < 0.05) and the assumption of sphericity was violated, data was corrected first, using Greenhouse-Geisser (ε < 0.75) and Shapiro-Wilk.

A repeated measures ANOVA design was applied to analyze CM response differences with different parameter settings. A significant main effect for stimulus (F(1.01, 9.12) = 28.71, *p* < 0.001, η^2^_p_ = 0.76) and a significant interaction effect for stimulus and polarity (F(1.04, 9.36) = 25.87, *p* < 0.001, η^2^_p_ = 0.74), both corrected with Greenhouse–Geisser, were found. Planned contrasts revealed that the click stimulus evoked a significantly larger CM amplitude than the 2 kHz tone burst stimulus (F(1, 9) = 28.49, *p* < 0.001, η^2^_p_ = 0.76) and the CE-chirp^®^ LS stimulus (F(1, 9) = 24.41, *p* < 0.001, η^2^_p_ = 0.73). CM amplitudes evoked by the 2 kHz tone burst stimulus and the CE-chirp^®^ LS stimulus did not differ significantly (F(1, 9) = 0.001, *p* > 0.05, η^2^_p_ = 0.00). Mean CM amplitudes are shown in Table 4.

The use of different HPFs, analyzed by repeated measures ANOVA, showed no significant different CM responses (F(1.01, 9.17) = 2.79, *p* < 0.05, η^2^_p_ = 0.24) for all three stimuli.

One-way ANOVAs were executed and showed significant differences between sexes for CM amplitudes evoked by a click and 3.3 Hz HPF in condensation (males = 2.57 nV, females = 1.37 nV, Δ = 1.20 nV, *p* < 0.05) and rarefaction polarity (males = 1.67 nV, females = 1.0 nV, Δ = 0.67 nV, *p* < 0.05). The absolute means per sexes are seen for each condition in Table 4. With respect to the other two stimuli, CM amplitudes evoked by 2 kHz tone bursts and CE-chirp^®^ LS were similar for both sexes; no significant differences were found (*p* > 0.05). Although not statistically significant, CM amplitudes evoked by a click stimulus showed larger amplitudes for both polarities when HPF was changed to 100 Hz. The use of different HPFs had larger consequences for the CM amplitudes evoked by click stimuli of males (largest SD = 1.15 nV) than females (largest SD = 0.93 nV), assessed by a one-way ANOVA (*p* < 0.05).

### 3.3. CM Latency and Duration

The planned contrasts showed no significant interactions between CM latency and CM duration when stimulus type or HPF was changed: CM latency (F(2, 18) = 0.39, *p* > 0.05, η^2^_p_ = 0.04) and CM duration (F(1.12, 10.11) = 4.13, *p* > 0.05, η^2^_p_ = 0.31) (repeated measures ANOVA).

With respect to differences between sexes, one-way ANOVAs showed no significant differences between the sexes for CM duration (*p* > 0.05), but a significant difference was seen for the CM latency evoked by a CE-chirp^®^ LS with HPF setting 100 Hz in both polarities, i.e., condensation (males = 0.82 ms, females = 0.43 ms, Δ = 0.56 ms, *p* < 0.05) and rarefaction (males = 1.06 ms, females = 0.52 ms, Δ = 0.54 ms, *p* < 0.05).

## 4. Discussion

The purpose of this study was to investigate the feasibility of using flexible silicon ET membrane electrodes instead of relatively invasive TT needle electrodes to obtain CMs. To our knowledge, this is the first study that explicitly describes the recording of CMs obtained with a flexible silicon TM electrode comparing clicks, 2 kHz tone bursts and CE-chirp^®^ LS stimuli. In addition to analyzing and proposing the most optimal stimulus parameter settings with respect to the different stimulus types, the HPF recording parameter was also analyzed for two different HPF settings during CM acquisition.

Firstly, results show that it is possible to obtain CMs with flexible silicon noninvasive TM electrodes. Secondly, the ECochG results reveal a 92%–100% presence of CMs when evoked by click stimuli, followed by a success rate of 75%–83% in response to 2 kHz tone bursts and 58%–63% to CE-chirp^®^ LS. Thirdly, data also clearly show that click stimuli evoke larger CM amplitudes compared to 2 kHz tone bursts and CE-chirp^®^ LS (see Figure 3). In contrast to prevailing stimulation rates that are clinically applied to obtain APs and/or wave V of the auditory brainstem (typically below 15 Hz), CMs do not require these lower stimulation rates since they do not depend on neural reactivity [1]. Although loss of auditory AP or peak V responses can occur by using a relatively high stimulation rate (87.1 Hz), the total measurement time can significantly be reduced, especially as clamped tube recordings are obligatory to confirm CMs [17]. In contrast to the two other sinusoidal stimuli, instantaneous fast activation of the basilar membrane in response to click stimuli enhance the CM amplitude, reflecting the total summation of the spatially activated OHC currents [3]. Since the power spectrum of a click stimulus mainly consists of relatively high frequencies, it is the basal part of the cochlea that is activated first [20], explaining the larger amplitudes due to the proximity of the recording TM electrode to the source [13].

It was also one of the aims of this study to analyze the surplus value of the chirp stimulus (see Appendix B) to evoke CMs. As far as we know, this is the only study using a TM-ECochG setup to obtain CM with chirps compared to conventional clicks and tone bursts. Although we are aware that the presentation level of the (shorter) click was higher in an absolute sense compared to the sinusoidal stimuli, based on the high success rates and the preceding loudness scaling task we performed in a pilot phase in order to equate the subjective loudness sensations of all three stimuli (following previous loudness scaling experiments investigating the effect of stimulus duration on loudness perception, e.g., [21]), the additional value of using a chirp stimulus seems to be limited, or at least not superior to a tone burst stimulus.

With respect to the differences between the sexes, the CM amplitudes of clicks were significantly larger for males than for females (Table 4). In contrast to the other two stimulus types, clicks showed larger standard deviations, regardless of the HPF setting. This inconsistency may be explained by the limited and low response amplitudes evoked by the other two stimuli, explaining the small standard deviation. Although both sexes showed larger standard deviations in CM amplitudes evoked by clicks, changing the HPF from 3.3 to 100 Hz only seems to affect males. However, in retrospect, it appeared that this was mainly due to data of two male subjects who showed extremely high CM amplitudes in 100 Hz HPF condition.

With respect to the HPF, CM morphologies as well as success rates did not seem to be influenced by the choice for a high or low HPF setting (3.3 vs. 100 Hz). Based on the spectral content of the CE-chirp^®^ LS stimulus (broadest frequency spectrum content), we expected that the difference between these two HPF might have some impact on the response morphology. In contrast, our data showed that the HPF did not really change the CMs for the BB-CE chirp, similar to the other two stimulus types. Although clinicians have been explicitly advocating for specific HPF settings in the past, e.g., 5 Hz [14,15] or 100 Hz [16,17], the present study did not show a clear preference for one of the two HPFs. This could be explained by the fact that (1) current EP-recording devices might have implemented higher quality filters, i.e., higher-order and more feasible modern digital filters, and (2) most of the previous ECochG studies were aimed at SP/AP potentials recordings instead of CMs; the use of widening the bandpass filter of the preamplifier for conventional hearing threshold ECochGs was needed to enable recognition of the DC component (SP) as well as the AC component (AP) [1]. Using higher HPFs may therefore significantly influence SP/AP recordings, but it may have less influence on CM morphology.

**Figure A1:**
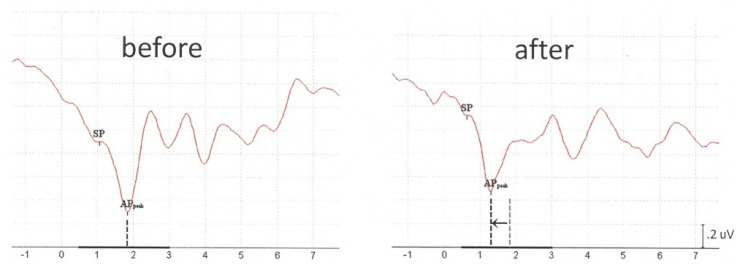
Typical SP/AP example of acute TM-ECochG within-subject recording before absorption of xylocaine showing a prolonged AP latency (left graph) and after absorption (right graph). Responses obtained by 90 dBnHL click stimulus in alternating polarity, stimulation rate 11.3/s and band pass filtered 100–3000 Hz.

Additionally, to record CMs as part of the TM-ECochG, we also acquired auditory neural responses (SP/APs) at lower stimulation rates (13.1 Hz) to confirm a correct hardware setup and interpretation of all components (data not shown). Although we were only interested in CMs, we would like to mention a phenomenon that might be relevant for clinicians who intend to use TM-ECochG for SP/AP recordings in clinical practice. In some subjects, it was very remarkable that absolute AP latencies were prolonged in comparison to TM-ECochG outcomes reported by other TM-ECochG studies, such as Grasel et al. [19]. Since all our subjects had normal hearing, we assumed that this peak latency delay was caused by a conductive component due to the presence of (too much) residual xylocaine in the ear canal. It is known that when vibrations are poorly transmitted between two materials with different densities, such as air and water, this mismatch can lead to a loss of intensity [22]. Drying the ear canal during preparation was manually performed with a cotton bud, but it might be that in some subjects the cotton bud had absorbed less xylocaine fluid during ear canal cleaning before auditory stimulation than in others. Though APs were not our research goal, we have repeated ECochG in a few subjects with significantly prolonged AP latencies, considering insufficient absorption of xylocaine in the ear canal. These repeated measurement results, acutely obtained in the same session, indeed showed normal AP latencies after thorough xylocaine absorption (see Appendix A, Figure A1: preabsorption vs. postabsorption). Although this requires more additional research, based on these limited data, we suggest clinicians at least to control for xylocaine dose absorption before acquiring SP/APs with TM electrodes.

The present research shows that application of the less invasive TM electrode in ECochG is feasible to obtain consistent CMs in clinical settings. Thus, in contrast to SP/AP recordings, CM has the advantage in that it allows the use of faster stimulation rates (e.g., 87.1 Hz, only limited by the length of stimuli) and, as a result, shorter measurement times. Clinical application of TM electrodes to record CMs might be limited to specific patient groups, but it has the advantage that it is less invasive compared to TT-ECochG. It has also been suggested that ET-ECochG might be a promising tool as the preoperative assessment in CI recipients because of its limited patient discomfort [10]. Future CM research should increase our knowledge of intracochlear structure preservation during cochlear implantation (CI), and hence, preoperative counseling or even side choice of implantation when structure preservation would show a substantial impact on postoperative hearing performance. Recent intraoperative CI studies already seem to point to a significant role of CM analyses [10]. Other applications of ET-ECochG are the assessment of patients suspected of ANSD [8] or of patients with mitochondrial disorders [7], with the goal to assess OHC functionality or the site of lesion.

In conclusion, this study contributed to the field of ECochG research with less invasive and more patient-friendly flexible silicon-shielded TM electrodes. The present study confirmed that this procedure can also be used to obtain CMs through efficient recording from the site of the tympanic membrane, provided that adequate preparation and experience of the clinician are met. Clicks generate the largest CM amplitude but, more importantly, show the highest success rate compared to the used sinusoidal stimuli and is therefore recommended as the preferred stimulus for confirming presence of CMs. The influence of using a low/high cut-off frequency of the HPF did not substantially change CM morphology. Stimulation and recording parameters for ET-ECochG are proposed to capture CMs with TM electrodes.

## Figures and Tables

**Table 1:** Stimulus parameters for the CM protocols per stimulus type (click, 2 kHz tone burst and CE-chirp^®^ LS).

Stimulus Parameters	Protocol 1	Protocol 2	Protocol 3
Stimulus type	Click	2 kHz tone burst	^1^ BB CE-chirp^®^ LS
Stimulus rate (Hz)	87.1	87.1	87.1
Polarity	^2^ Rare & ^3^ cond	Rare & cond	Rare & cond
Duration (ms)	0.1	1.5	5
Envelope	-	1-1-1 cycle	-
Intensity (dBnHL)	100	80	80

^1^ BB = broadband, ^2^ rare = rarefaction, ^3^ cond = condensation.

**Table 2:** Recording parameters for all CM protocols.

Recording Parameter	CM Protocols
Stimulation	Monaural
Type electrode	Tympanic membrane (TM) electrode (Sanibel, Denmark)
Electrode positioning	Vertical montage
Averaging (total)	2 × 1500 (3000)
^1^ HPF–^2^ LPF (Hz)	3.3–3000 or 100–3000
Amplification	100,000×

^1^ HPF = high-pass filter, ^2^ LPF = low-pass filter.

**Table 3:** Success rates of cochlear microphonics (CM) in response to each stimulus type.

^1^ HPF	^2^ LPF	CM Click	CM 2 kHz Tone Burst	CM CE-Chirp^®^ LS
3.3 Hz	3000 Hz	22/24 (92%)	20/24 (83%)	14/24 (58%)
100 Hz	3000 Hz	24/24 (100%)	18/24 (75%)	15/24 (63%)

^1^ HPF = high-pass filter, ^2^ LPF = low-pass filter.

**Table 4:** Means and standard deviations of CM amplitudes (in nV) evoked by click, 2 kHz tone burst and CE-chirp^®^ LS.

Stimulus Type	Polarity	^1^ HPF	Females CM Amplitude Mean (^4^ SD)	Males CM Amplitude Mean (SD)
Click	^2^ Cond ^3^ Rare	3.3 Hz 100 Hz 3.3 Hz 100 Hz	1.37 (0.93) 1.32 (0.79) 1.00 (0.67) 0.94 (0.60)	2.57 (1.15) 1.68 (0.95) 1.67 (0.82) 1.23 (0.79)
2 kHz tone burst	Cond Rare	3.3 Hz 100 Hz 3.3 Hz 100 Hz	0.12 (0.05) 0.15 (0.05) 0.15 (0.07) 0.14 (0.05)	0.13 (0.04) 0.11 (0.03) 0.12 (0.04) 0.12 (0.02)
CE-chirp^®^ LS	Cond Rare	3.3 Hz 100 Hz 3.3 Hz 100 Hz	0.23 (0.12) 0.20 (0.11) 0.21 (0.11) 0.21 (0.10)	0.16 (0.08) 0.12 (0.04) 0.16 (0.08) 0.19 (0.06)

^1^ HPF = high-pass filter, ^2^ cond = condensation, ^3^ rare = rarefaction, ^4^ SD = standard deviation.

## Data Availability

The datasets generated for this study are available on request to the corresponding author.

## Appendix B

For a description of chirp stimuli used in the present study, we refer to Elberling and Don [23]: the main principle of using the broad band chirp is to stimulate the basilar membrane simultaneously due to the presentation of a broad band stimulus that contains multi-frequencies [20,23]. Typically, chirp stimuli consist of multi-frequencies that are based on a model covering the traveling wave times in the cochlea. For the present study, a BB CE-chirp^®^ LS was used that was designed so that the zero point on the time axis (t0) corresponds to the frequency of 10 kHz in the underlying delay model. This point corresponds to the starting point (t0) of the recording in the Interacoustics EP25 device, functioning as the temporal reference for every EP recording. Using this chirp, the 10 kHz component is delayed by 1.5 ms to arrive at the start at 0 ms, thus resulting in prolonged latencies of the chirp for all frequencies [24]. For other examples of chirp models, we would like to refer to the excellent work of Fobel and Dau [25], who gave an overview of different chirp models that have been developed. Chertoff et al. [20] have clearly described the temporal and spectral differences between clicks and chirps in time and frequency domain. They have reported that small differences in spectral contents were found between these two stimuli below 4 kHz with the click stimulus having more energy than the chirp stimulus.
